# Visual pathology reports for improved collaboration at multidisciplinary head and neck tumor board

**DOI:** 10.1002/hed.27926

**Published:** 2024-08-29

**Authors:** Carly Fassler, Pratyusha Yalamanchi, Marina Aweeda, Julie Rezk, Barbara Murphy, Natalie A. Lockney, Ryan Whitaker, Ryan Rigsby, Joseph Aulino, Emily Hosokawa, Mitra Mehrad, Kim Ely, James S. Lewis, Evan Derman, Ed LaHood, Sarah L. Rohde, Robert J. Sinard, Eben L. Rosenthal, Michael C. Topf

**Affiliations:** ^1^ Department of Otolaryngology – Head and Neck Surgery Vanderbilt University Medical Center Nashville Tennessee USA; ^2^ Department of Oral & Maxillofacial Surgery Vanderbilt University Medical Center Nashville Tennessee USA; ^3^ Department of Hematology and Oncology Vanderbilt Ingram Cancer Center Nashville Tennessee USA; ^4^ Department of Radiation Oncology Vanderbilt Ingram Cancer Center Nashville Tennessee USA; ^5^ Department of Radiology Vanderbilt University Medical Center Nashville Tennessee USA; ^6^ Department of Hearing and Speech Sciences Vanderbilt University Medical Center Nashville Tennessee USA; ^7^ Department of Pathology, Microbiology & Immunology Vanderbilt University Medical Center Nashville Tennessee USA; ^8^ Department of Laboratory Medicine and Pathology Mayo Clinic Phoenix Arizona USA; ^9^ MedReality, Thyng LLC Chicago Illinois USA; ^10^ Vanderbilt University School of Engineering Nashville Tennessee USA

**Keywords:** 3D imaging, head and neck cancer, multidisciplinary communication, tumor boards, visual pathology

## Abstract

**Purpose:**

Multidisciplinary tumor boards (TB) are the standard for discussing complex head and neck cancer cases. During TB, imaging and microscopic pathology is reviewed, but there is typically no visualization of the resected cancer.

**Methods:**

A pilot study was conducted to investigate the utility of visual pathology reports at weekly TB for 10 consecutive weeks. Faculty‐level participants completed a pre‐survey and post‐survey to assess understanding of resected cancer specimens.

**Results:**

Providers (*n* = 25) across seven medical specialties completed pre‐survey and post‐survey. Following intervention, providers reported significant improvement in understanding of anatomic orientation of the specimen and sites of margin sampling (mean 47.4–96.1, *p* < 0.001), ability to locate the site of a positive margin (mean 69.5–91.1, *p* < 0.001), and confidence in treatment plans created (mean 69.5–89.2, *p* < 0.001) with the addition of visual pathology reports.

**Conclusions:**

Visual pathology reports improve provider understanding of resected cancer specimens at multidisciplinary TB.

## 1 INTRODUCTION

Multidisciplinary tumor board (TB) meetings are the standard of care for the discussion of complex oncologic care for patients across all solid malignancies.^1^ Head and neck cancer (HNC) in particular represents a uniquely heterogeneous group of neoplasms arising from various anatomic subsites, each with distinct etiologies, diagnostic approaches, and treatment paradigms. This diversity of cancer subtypes, coupled with the significant morbidity that often occurs as a result of treatment in this region, demands special multidisciplinary collaboration to optimize both oncologic and functional outcomes.

Given the complex, three‐dimensional (3D) anatomy and multiple tissue types present in the head and neck, orientation and margin analysis of resected cancer specimens, as well as the subsequent communication of final pathology results back to the multidisciplinary cancer care team can be challenging.^2^ Following surgical resection, the pathology assistant (PA) or resident physician typically analyzes the specimen and communicates the gross measurements of the specimen, tumor characteristics, and sites selected for margin analysis via a written report.^3^, ^4^, ^5^ This is then finalized by the attending pathologist during microscopic slide analysis, which often occurs days to weeks following the surgical resection. As the 3D structure of the oncologic resection is disrupted during the grossing process, this may pose a challenge when there is a need to return to the main specimen to take additional sections, clarify final margin status, or for reference during subsequent adjuvant treatment planning and multidisciplinary care discussions. There is currently no available visual data of the 3D structure of the resected surgical specimen to help providers understand the anatomic structure and orientation of the specimen or the precise sites of margin sampling. This introduces a potential disconnect for providers (all members of the TB other than the surgeon) who have never seen the resected cancer, and must participate in multidisciplinary care discussions with only the visual aids of radiologic imaging and microscopic pathology slides.

In an increasingly digital world, 3D innovations are numerous and have become a well‐established avenue of research in oncology.^6^, ^7^, ^8^, ^9^, ^10^ Prior work has demonstrated that ex vivo optical 3D scanning and virtual 3D specimen mapping is a valuable communication tool for surgeons and pathologists for intraoperative frozen section analysis,^11^, ^12^, ^13^, ^14^, ^15^, ^16^ and has the ability to provide a visual representation of the processing of pathologic specimens.^17^, ^18^ However, the use of visual pathology reports for the purpose of enhancing communication among the multidisciplinary care team remains understudied. We aimed to build upon our prior work and establish the broader use of visual pathology reports as a communication tool at multidisciplinary head and neck TB. This 10‐week intervention piloted the use of visual pathology reports as a supplement to the standard of care case presentation at TB. Pre‐ and post‐implementation survey data was collected to assess provider perception of the utility and impact of visual pathology reports at head and neck TB across various specialties.

## 2 METHODS

### 2.1 Study population

This prospective study was approved by the Vanderbilt University Medical Center Institutional Review Board (IRB #231602). At our institution, virtual TB meetings are held weekly via videoconference (Microsoft Teams, Redmond, WA). Healthcare providers of all medical specialties present at weekly head and neck TB were invited to participate in this study. Team members provided written consent prior to participation. Specialties represented include head and neck surgical oncology, medical oncology, radiation oncology, pathology, radiology, speech‐language pathology, and dentistry.

### 2.2 Pre‐survey

A pre‐survey was distributed to providers of all specialties about their opinions on current tools available for case presentation at multidisciplinary TB. Survey responses were set on a scale of 0–100, 0 being “Strongly Disagree” and 100 being “Strongly Agree” to each proposed statement. Survey data were collected and managed using a secure electronic data capture tool (REDCap, Fort Lauderdale, FL) hosted at Vanderbilt University Medical Center. Pre‐surveys were distributed to providers via email by a research team member. The pre‐survey instrument is included in Figure S1, Supporting Information.

### 2.3 3D scanning and specimen mapping

**FIGURE 1:**
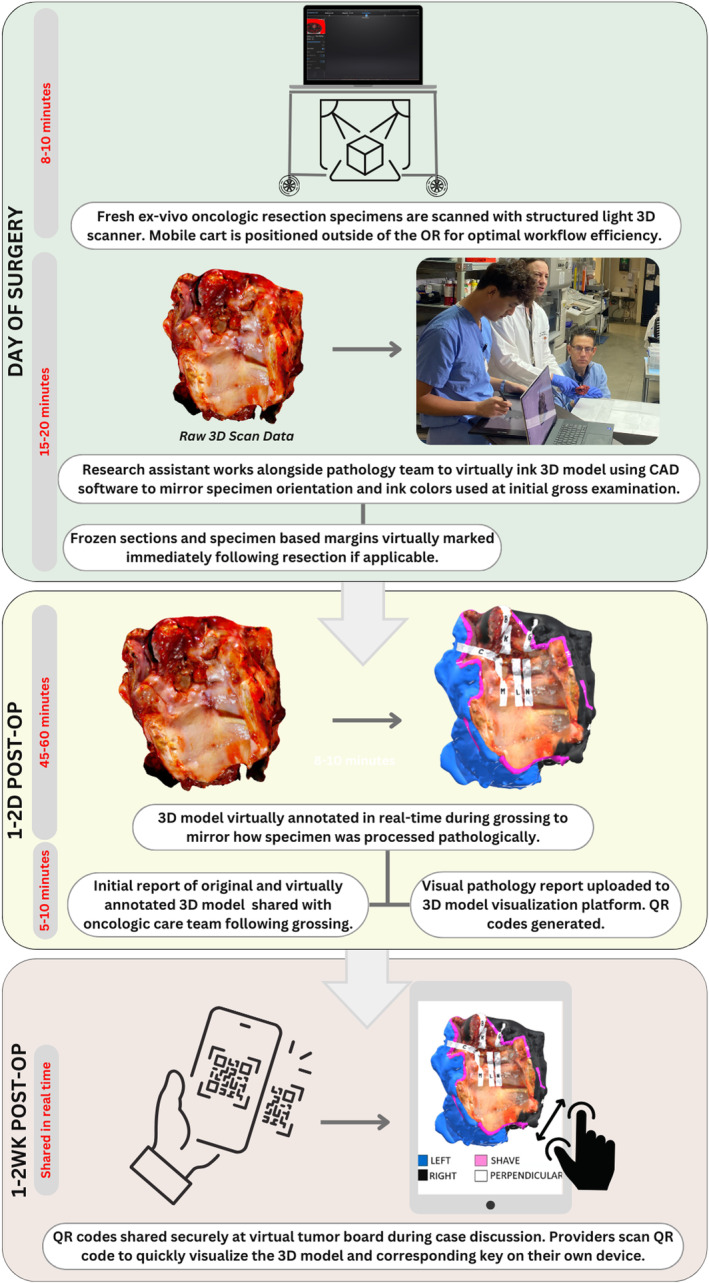
Study protocol: Overview of study workflow. [Color figure can be viewed at wileyonlinelibrary.com]

A 3D scanning and virtual specimen mapping protocol has been integrated into the surgical pathology workflow at our institution since October 2021. We have previously published our 3D scanning and specimen mapping technique to create visual pathology reports.^11^, ^12^, ^18^, ^19^ A schematic detailing the protocol for this method can be seen in Figure 1. 3D scans of resected head and neck primary tumor surgical specimens were obtained by a research team member based on availability. 3D scans of locally advanced oncologic resections were obtained preferentially when possible given inherent challenges with anatomic orientation, though as many cases as possible were 3D scanned. 3D scans were virtually annotated alongside the pathology team to mirror grossing of the main specimen. All patients provided written consent for the 3D scanning of their surgical specimens and to have their visual pathology report data saved in a secure biorepository (VUMC IRB #221597).

### 2.4 Study intervention

**FIGURE 2:**
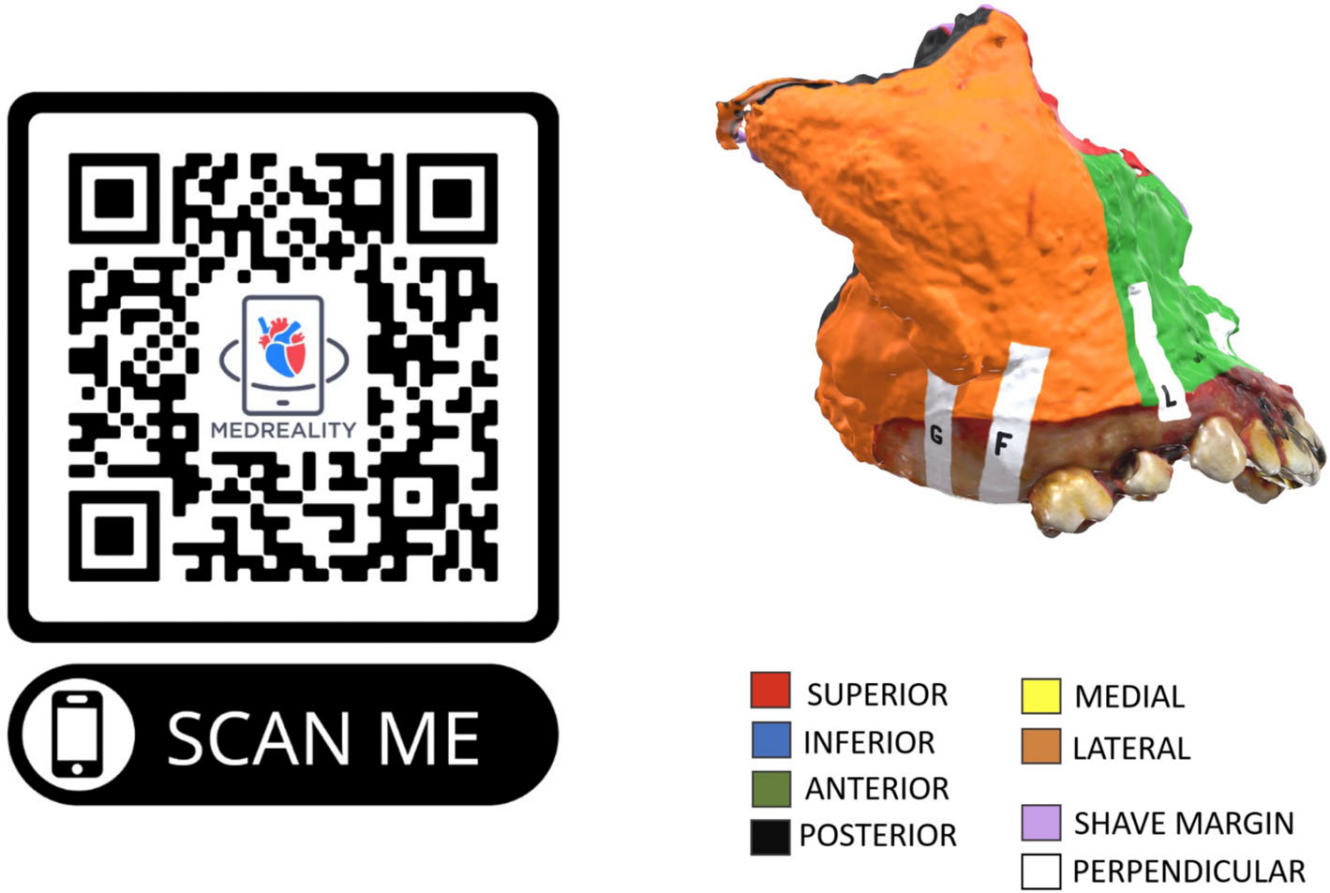
Visual pathology report: Representative QR code (left) and visual pathology report (right) for case #6, of a maxillectomy for adenoid cystic carcinoma of the maxillary sinus. [Color figure can be viewed at wileyonlinelibrary.com]

**FIGURE 3:**
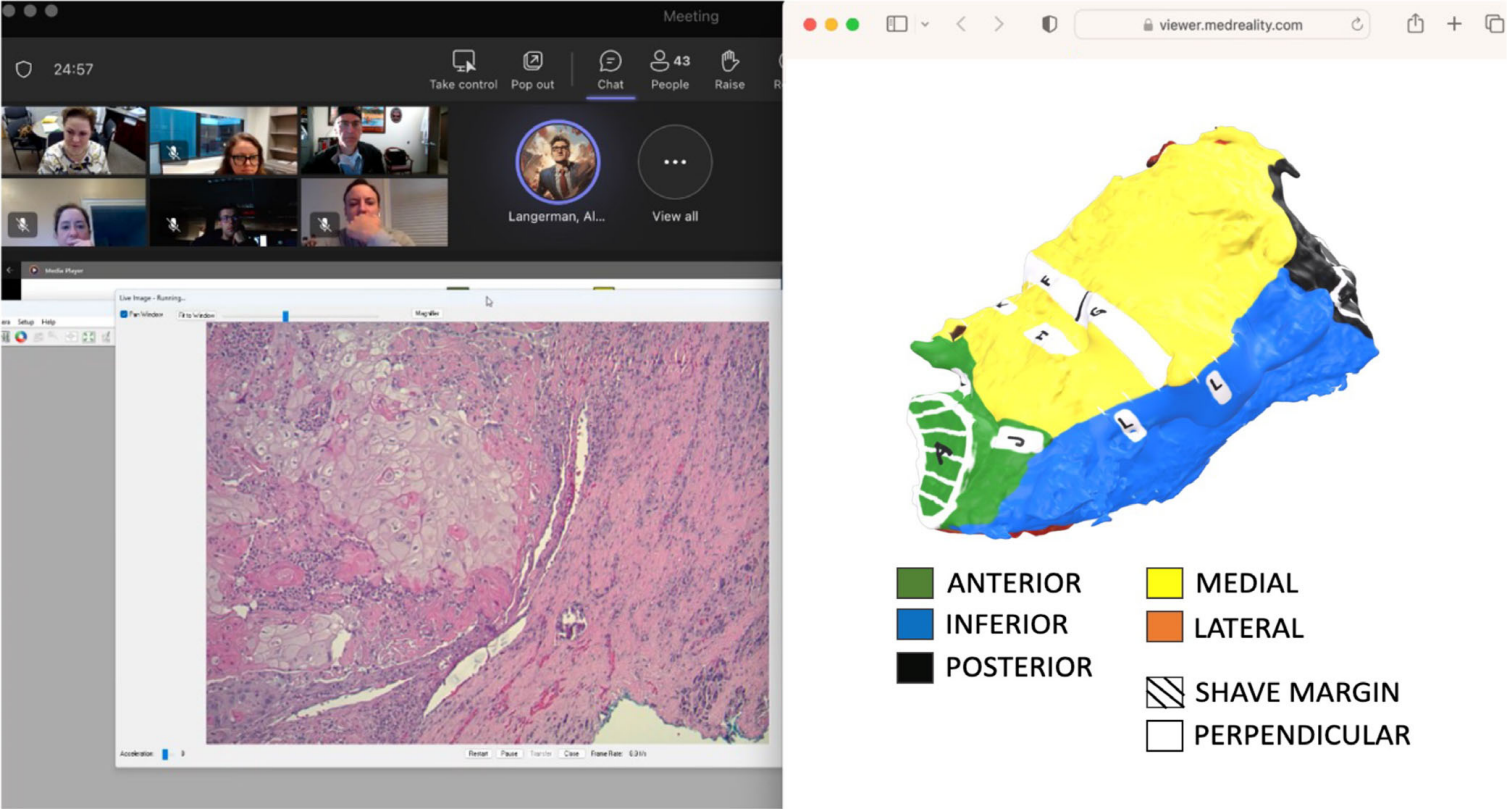
Tumor board discussion with visual pathology report: Virtual tumor board discussion among providers regarding final pathology for case #5, an oral cavity composite resection for p16+ squamous cell carcinoma of the retromolar trigone. The microscopic slide (left) for the margin of interest is shown alongside the 3D model (right) demonstrating the exact location of this margin with corresponding color‐coded key. [Color figure can be viewed at wileyonlinelibrary.com]

One visual pathology report was presented each week at virtual head and neck TB between December 1, 2023 and March 1, 2024 for a total of 10 consecutive weeks. Cases were selected based on need for adjuvant treatment discussion. At our multidisciplinary TB, surgeons select resected cases to discuss for adjuvant treatment discussion. Not all postoperative surgical cases are discussed. Visual pathology reports were uploaded to a novel 3D model visualization platform (MedReality, Chicago, IL) that allows users to view 3D models across a variety of technologies, including smartphone, tablet, computer, and augmented reality (AR) and virtual reality (VR) devices. A color‐coded key was uploaded and displayed beneath the interactive model. For optimal integration with the time‐constrained environment of multidisciplinary TB at a large academic institution, QR codes were generated and screen‐shared to allow providers to access the fully interactive virtual specimen map and key on their own device. A representative QR code with its corresponding case can be seen in Figure 2. QR codes with each corresponding 3D model for the remaining cases are available in Figures S2–S10. A direct link to the model was also provided in the meeting chat for providers who may not have had a separate device available to scan the QR code. A case discussion among providers regarding final pathology using the 3D model in real‐time at TB is shown in Figure 3.

### 2.5 Case characteristics

Case characteristics were extracted from the electronic medical record (EMR) for each patient. Chart review was performed to determine procedure type, prior history of radiation therapy (RT), and adjuvant treatment recommendations. Final pathology reports were reviewed to determine anatomic location, tumor histology, final margin status, and pathologic stage. All reported staging was based on the eighth edition of the American Joint Committee on Cancer (AJCC 8th ed.) staging system. Surgical margin status was based on our institutional standards. Positive margins were defined as invasive tumor at the ink, close margins as <5 mm to invasive tumor, and negative margins as ≥5 mm from invasive tumor.

### 2.6 Post‐survey

Following the 10‐week intervention, a post‐survey was distributed to pre‐survey participants about their opinions on the utility of visual pathology reports in addition to the currently available tools at weekly head and neck TB. Survey responses were set on a scale of 0–100, 0 being “Strongly Disagree,” 50 being “Neutral” and 100 being “Strongly Agree” to each proposed statement. Post‐surveys were created using REDCap and were distributed to providers via email by a research team member. The post‐survey instrument is included in Figure S11.

### 2.7 Statistical analysis

All statistical analyses were conducted using SPSS version 22.0 (SPSS Inc., Chicago, IL). Data was entered using a participant number to ensure anonymity of individual responses. Examination of histograms, skewness and kurtosis, and Kolmogorov–Smirnov tests confirmed normality of data. Therefore, paired sample *t* tests were used to evaluate the differences between pre‐ and post‐intervention survey scores. Comparison of survey respondent group characteristics (i.e., faculty specialty and years in practice) was performed using one‐way analysis of variance if the variable was normally distributed and using Kruskal–Wallis tests if the variable was non‐normally distributed. Alpha was set at 0.05 and all *p*‐values were two‐sided.

## 3 RESULTS

### 3.1 Case characteristics

One case was presented at each weekly TB for 10 consecutive weeks. Cases with an available visual pathology report that were submitted for discussion at TB by a provider were considered for discussion. Anatomic subsites included oral tongue (*n* = 2), floor of mouth (*n* = 2), retromolar trigone (*n* = 1), buccal mucosa (*n* = 1), supraglottic larynx (*n* = 1), subglottic larynx (*n* = 2), and maxillary sinus (*n* = 1). Histologic analysis revealed non‐HPV related squamous cell carcinoma (*n* = 7) and adenoid cystic carcinoma (*n* = 3). All cases were pathologic stage T3 (*n* = 3) or T4 (*n* = 7). Discussed cases had final margin status of positive (*n* = 4), close (*n* = 4), or negative (*n* = 2) margins. Perineural invasion (PNI) was present in five cases, lymphatic‐vascular invasion (LVI) in one case, and neither was present in four cases. Final TB recommendations were adjuvant chemoradiation (*n* = 4), adjuvant RT alone (*n* = 3), and surveillance (*n* = 3).

In cases of close (cases 1, 2, and 8) or positive (cases 3, 5, 6, and 7) margins, the visual pathology report was used to view the exact anatomic location of the area closest to or containing invasive tumor. In cases of positive margins, the visual pathology report provided surgeons and pathologists with a visual model of the resection to confirm that no additional tumor bed‐based margins superseded the positive specimen margin. For both close and positive margin cases, the visual pathology report fostered discussion among radiation oncologists regarding directed RT planning. For example, in case 1 there was a close margin (2 mm from invasive tumor) at the left anterolateral soft tissue margin (labeled C). Providers utilized the model to visualize the laryngectomy specimen and determined that although there was a close margin at this location, additional RT was not feasible due to prior treatment of this area to 70 Gy. Additional surgical and pathologic characteristics as well as final tumor board recommendations for each case are shown in Table 1.

**TABLE 1:** Case characteristics.

Anatomic location	Procedure	Cancer histology	pTN stage	Margin status	Adjuvant tx recommendation	History of prior RT
Supraglottic larynx	TL	SCCa	T4aN0	Close	Surveillance^a^	Y
FOM	OCCR	SCCa	T3N0	Close	CRT	N
Buccal mucosa	Buccal resection	SCCa	T4aN3b	Positive	CRT	N
Subglottic larynx	TL	ACC	T4aN0	Negative	RT	N
Retromolar trigone	OCCR	SCCa	T4aN1	Positive	CRT	N
Maxillary sinus	Maxillectomy	ACC	T4bN0	Positive	CRT	N
Subglottic larynx	TL	ACC	T4aN0	Positive	RT	N
Lateral tongue	Partial glossectomy	SCCa	T3N0	Close	RT	N
FOM	Anterior composite	SCCa	T4aN0	Negative	Surveillance	N
Lateral tongue	Partial glossectomy	SCCa	T3N2a	Negative	Surveillance	Y

Abbreviations: ACC, adenoid cystic carcinoma; CRT, chemoradiotherapy; FOM, floor of mouth; OCCR, oral cavity composite resection; pTN, pathologic tumor, nodal staging; RT, radiotherapy; SCCa, squamous cell carcinoma; TL, total laryngectomy; tx, treatment.

^a^ Patient had already undergone CRT twice, precluding additional radiation therapy to the neck.

### 3.2 Survey data

**FIGURE 4:**
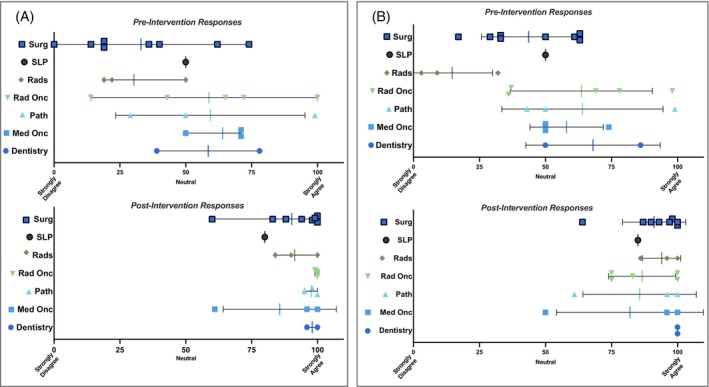
Pre‐ and post‐intervention survey responses for questions 3 (A) and 5 (B) by medical specialty. Question 3: *The 3D specimen map in addition to* the current tools used for discussion at head and neck tumor board enhances understanding of the anatomic orientation and sites of margin sampling during pathologic processing. Question 5: I feel confident in locating the site of a positive margin *using the 3D specimen map* in addition to the current tools available at head and neck tumor board. Italics represents post‐intervention survey question phrasing. Surg, head and neck surgeon; SLP, speech language pathologist; Rads, radiologist; Rad Onc, radiation oncologist; Path, pathologist; Med Onc, medical oncologist. Mean = vertical colored line, standard deviation = horizontal black line. [Color figure can be viewed at wileyonlinelibrary.com]

A total of 25 providers from head and neck TB across seven specialties were included. All faculty‐level providers that attend multidisciplinary head and neck TB at a single institution completed the pre‐survey and post‐survey (100% response rate). Survey respondent characteristics are shown in Table 2. Prior to the implementation of visual pathology reports at TB, providers were neutral with the statement that the current tools available at TB for understanding anatomic orientation and sites of margin sampling during pathologic processing were adequate, with a mean response of 47.4 (SD 27.1) Following intervention, mean response to this statement increased to 96.1 (SD 8.2) (95% CI [37.0–60.3], *p* < 0.001) (Figure 4). Similarly, the pre‐intervention mean provider confidence in locating the site of a positive margin using the current tools available was 50.5 (SD 24.9). Following intervention, mean response to this statement increased to 91.1 (SD 12.8) (95% CI [28.9–52], *p* < 0.001) (Figure 4). At the conclusion of the study, providers agreed that the visual pathology reports presented at head and neck TB were high quality (mean response 97.2, SD 4.2) and could be integrated into the workflow of head and neck TB (mean response 95.9, SD 9.1). Pre‐ and post‐intervention results for all survey questions are summarized in Table 3. There was no significant difference in survey responses when analyzed by medical specialty or number of years in practice (Table S1).

**TABLE 2:** Survey respondent characteristics.

Number of respondents^a^, *n* (% of multidisciplinary tumor board members)	25 (100)
Medical specialty	
Head and neck surgeon, *n* (% of division)	8 (100)
Radiation oncologist, *n* (% of division)	5 (100)
Radiologist, *n* (% of division)	3 (100)
Surgical pathologist, *n* (% of division)	3 (100)
Medical oncologist, *n* (% of division)	3 (100)
Dentist, *n* (% of division)	2 (100)
Speech language pathologist, *n* (% of division)	1 (100)
Years in practice, mean (SD)	20.2 (11.4)

^a^ No loss of respondents from pre‐survey to post‐survey.

**TABLE 3:** Pre‐ and post‐intervention survey results.

Survey statements^a^	Pre‐intervention survey responses (*n* = 25), mean (SD)	Post‐intervention survey responses (*n* = 25), mean (SD)	95% confidence interval	*p*‐value
The surgery, pathology, medical oncology, radiation oncology, and radiology teams are able to easily communicate and understand tumor characteristics for treatment planning at head and neck tumor board	68.5 (21)	97.2 (4)	20.3–37.2	<0.001
*The 3D specimen map in addition to* the current tools used for discussion at head and neck tumor board (i.e., operative reports, pathology reports, verbal discussions among the multidisciplinary team, pre‐ and postoperative imaging) enhances understanding of the tumor size and characteristics	64.5 (22.7)	92.8 (11.4)	17.7–38.9	<0.001
*The 3D specimen map in addition to* the current tools used for discussion at head and neck tumor board (i.e., operative reports, pathology reports, verbal discussions among the multidisciplinary team, pre‐ and postoperative imaging) enhances understanding of the anatomic orientation and sites of margin sampling during pathologic processing	47.4 (27.1)	96.1 (8.2)	37.0–60.3	<0.001
I feel confident in the treatment plans created using *the 3D specimen map in addition to* the current tools available at head and neck tumor board	69.5 (21.8)	89.2 (14.1)	9.4–30.1	<0.001
I feel confident in locating the site of a positive margin using *the 3D specimen map in addition to* the current tools available at head and neck tumor board	50.5 (24.9)	91.1 (12.8)	28.9–52.2	<0.001
The 3D specimen maps presented at head and neck tumor board are high quality^b^	–	97.2 (4.2)	–	–
I believe that 3D specimen maps can be integrated into the workflow of head and neck tumor board^b^	–	95.9 (9.1)	–	

^a^ Italicized text refers to the additional text added to the post‐intervention survey questions.

^b^ Additional post‐survey questions not asked in the pre‐survey.

## 4 DISCUSSION

In the present study, we surveyed faculty‐level head and neck surgical oncologists, pathologists, radiation oncologists, medical oncologists, radiologists, speech language pathologists, and dentists to assess the degree to which they consider that the current tools available during TB are adequate to understand the anatomic orientation of the resected specimen and sites of margin sampling for complex head and neck cancer cases. The responses varied widely, with most respondents disagreeing or feeling neutral toward this statement. A similar pattern of responses was observed when respondents were asked about their confidence in locating the site of a positive margin using currently available tools for discussion at TB. To address this knowledge gap, we implemented a 10‐week intervention in which visual pathology reports were shared to improve communication at weekly head and neck TB. Post‐intervention survey responses revealed an increase in overall confidence by providers of all specialties in orienting anatomic specimens and understanding sites of margin sampling with the addition of visual models. These findings were durable across faculty specialty and years in practice.

Multidisciplinary TB discussions are the standard for the coordinated care of complex oncologic patients and provide a platform for treatment planning and collaboration among the entire cancer care team. However, there is little existing research on improving methods of communication at TB, specifically as it relates to tools available for discussing anatomic orientation and sites of margin sampling in complex oncologic resections. The current methods of communication for discussing complex pathology and margin status should be improved, as evidenced by the lack of consensus among providers in our pre‐study survey surrounding these areas. Prior studies have demonstrated the potential for error in communication of intraoperative frozen section diagnosis^20^, ^21^ and interpretation of final pathology reporting.^22^, ^23^, ^24^, ^25^ One study found that in up to 30% of cases, surgeons misunderstood final pathology reports,^22^ while another demonstrated that 27/29 pathology reports reporting final positive margins were reclassified as clear (*n* = 26) or close (*n* = 1) following TB discussion.^26^ While various methods have been proposed for improving the readability of pathology reports with organization and synoptic reporting, few studies have investigated the utility of a visual aid for final pathology reporting or TB discussion of complex cases. One report investigated the use of image fusion to provide a visual representation of the case at the time of TB discussions,^27^ while another examined the utility of sharing radiation dose distribution maps in the EMR.^28^

Although several providers of varying specialties are involved in cancer care, the majority of the time only the surgeon and the PA or resident physician who processes the specimen actually visualize the resected cancer and its orientation. Although surgeons are occasionally present in the gross room and indicate particular margins they are concerned about to the prosector, this is not necessarily routine. Therefore, the individual who processes the case is the only provider who truly sees exactly where each margin is taken. The prosector provides written text describing the specimen and a list of margins that must be correlated to cassettes submitted for microscopic analysis by the attending pathologist. Following microscopic analysis, the final pathology report is then released to the patient's chart for interpretation by the multidisciplinary cancer care team. With no visual representation of the 3D orientation of the specimen or sites of margin sampling available for review by the wider cancer care team, errors in report interpretation, multidisciplinary discussions, and adjuvant treatment planning can occur. The frequency of specimen orientation and margin sampling errors is challenging to study and quantify. However, in our experience using visual pathology reports routinely for postoperative communication between surgeons and pathologists, these errors do occur, a minority, but not insignificant, amount of the time.

This issue is particularly relevant in patients that require adjuvant radiation, wherein the treating radiation oncologist, who has not directly visualized the tumor, determines the postoperative target volumes based on the diagnostic images, operative report, written pathology report, and verbal conversations with the surgeon and/or pathologist. Sites of positive margins may often be selected for a higher radiation dose, or boost. The lack of a 3D visual aid in specifying margin locations for boost delineation makes it difficult for the radiation oncologist to understand the exact anatomic areas with close or positive margins – which may lead to a less targeted postoperative radiation field and result in increased side effects and poorer outcomes.^8^, ^29^ Our team is actively investigating the use of 3D‐printed models of oncologic resections in RT treatment planning (NCT#05743569). Ultimately, changes in treatment course and radiation field or dose carry long term implications for functional speech and swallow outcomes in patients with head and neck cancer.^30^ This is particularly significant as swallowing function remains one of the strongest predictors of quality of life in head and neck cancer survivors.^31^ It is reasonable to hypothesize that increased specificity of RT treatment planning or changes in adjuvant treatment recommendations through the use of visual pathology reports may positively impact swallowing outcomes in head and neck cancer survivors, though this is an area requiring further investigation. This study further envisions a future in which visual pathology reports are available for review with all complex head and neck cancer cases, to improve multidisciplinary understanding and communication among all providers present at TB discussions.

There are several limitations to this study. First, the largest pitfall to this method is that it is currently not universally transferrable or available at many institutions. Specialized equipment is required to perform 3D scanning and virtual specimen mapping. The time required to train research team members in 3D scanning and mapping and the time to create visual pathology reports on a routine basis must be acknowledged. To address this concern, we have developed a protocol for training new team members on this protocol efficiently and have streamlined our method with the current surgical workflow. We work to keep time for creation of a single pathology report under 1‐h total by utilizing a mobile 3D scanning cart that can be positioned conveniently outside of the operating room or pathology lab, annotating models in real‐time alongside the prosector to minimize additional time added to grossing, and investigating new technologies to make creation easier and faster.

We have also considered the idea of accomplishing similar goals with 2D annotated gross photographs, which may be more universally available. This could be accomplished by taking 2D images of the resected cancer specimen and utilizing a commercially available software program for annotation. Similar methods using margin tags and anatomical templates to diagram areas of margin sampling in a specimen‐based approach have been proposed.^32^, ^33^ The drawback to the use of 2D images is that they are not interactive or dynamic, and viewers are not be able to see the entire 3D representation of the specimen at the time of resection and pathologic grossing. The MedReality 3D model visualization platform allows us to instantly upload our virtual models and generate a QR code that can be distributed quickly and easily.

Response bias may have occurred given that we only surveyed individuals within our institution. We attempted to mitigate this bias by using secure REDCap surveys that were distributed to providers individually via email by a research assistant who is not a regular member of the care team. The independent research team member reviewed survey responses and de‐identified them for analysis to protect individual survey responses.

The QR codes and visual pathology reports were only available at the time of tumor board in this study. Future directions of this work include incorporating the models into the EMR, which would allow providers to view the 3D model within the final pathology report as a visual gross pathology representation. To further build out the infrastructure of this method, we hope to develop custom software for the seamless creation of visual pathology reports by pathology providers that can be automatically integrated into the EMR. We additionally are interested in exploring other multimedia integrations, including incorporating digital pathology slides into the 3D models that would open when a particular margin is clicked. Further investigations related to this work include continuing to explore the use of visual pathology reports for adjuvant RT planning and to increase patient knowledge about their cancer through patient‐centered pathology. Retrospective correlation between visual pathology reports and preoperative imaging may provide insights into better predicting tumor margins on preoperative imaging, which warrants additional investigation. Further studies are required to demonstrate the potential impact of visual pathology reports on the health‐system and patient clinical oncologic outcomes.

## CONFLICT OF INTEREST STATEMENT

The authors declare no conflicts of interest.

## ETHICS STATEMENT

This study was reviewed and approved by the Vanderbilt University Medical Center Institutional Review Board (IRB #231602). Written informed consent was obtained from all patients included in this study.

## Supporting information


**Figure S1.** Pre‐survey administered to tumor board participants prior to intervention.
**Figure S2.** Total laryngectomy specimen for laryngeal squamous cell carcinoma.
**Figure S3.** Oral cavity composite resection for squamous cell carcinoma of the floor of mouth.
**Figure S4.** Buccal resection for squamous cell carcinoma of buccal mucosa.
**Figure S5.** Total laryngectomy specimen for laryngeal squamous cell carcinoma.
**Figure S6.** Oral cavity composite resection for squamous cell carcinoma of the retromolar trigone.
**Figure S7.** Total laryngectomy specimen for laryngeal adenoid cystic carcinoma.
**Figure S8.** Partial glossectomy for squamous cell carcinoma of the oral tongue.
**Figure S9.** Anterior composite resection for squamous cell carcinoma of the floor of mouth.
**Figure S10.** Partial glossectomy for squamous cell carcinoma of the oral tongue.
**Figure S11.** Post‐survey administered to tumor board participants following intervention.

## Data Availability

The data that support the findings of this study are available in Supporting Information of this article.
