# Supplementary material for: Visual pathology reports for improved collaboration at multidisciplinary head and neck tumor board
Source: Head Neck. 2024 Aug 29;47(2):452–62. doi: 10.1002/hed.27926 (PMC11717968; doi:10.1002/hed.27926)
Supplement: Supplementary file 1 — Figure S1. Pre‐survey administered to tumor board participants prior to intervention. Figure S2. Total laryngectomy specimen for laryngeal squamous cell carcinoma. Figure S3. Oral cavity composite resection for squamous cell carcinoma of the floor of mouth. Figure S4. Buccal resection for squamous cell carcinoma of buccal mucosa. Figure S5. Total laryngectomy specimen for laryngeal squamous cell carcinoma. Figure S6. Oral cavity composite resection for squamous cell carcinoma of the retromolar trigone. Figure S7. Total laryngectomy specimen for laryngeal adenoid cystic carcinoma. Figure S8. Partial glossectomy for squamous cell carcinoma of the oral tongue. Figure S9. Anterior composite resection for squamous cell carcinoma of the floor of mouth. Figure S10. Partial glossectomy for squamous cell carcinoma of the oral tongue. Figure S11. Post‐survey administered to tumor board participants following intervention. [file HED-47-452-s001.pdf]

Supplementary Table 1: Impact of Survey Respondent Characteristics on Survey Results						
	The 3D specimen maps presented at head & neck tumor board are high quality. (p-value)	I believe that 3D specimen maps can be integrated into the workflow of head & neck tumor board. (p-value)	The 3D specimen map in addition to the current tools used for discussion at head & neck tumor board (i.e. operative reports, pathology reports, verbal discussions among the multidisciplinary team, pre and post-operative imaging) enhances understanding of the tumor size and characteristics. (p-value)	The 3D specimen map in addition to the current tools used for discussion at head & neck tumor board (i.e. operative reports, pathology reports, verbal discussions among the multidisciplinary team, pre and post-operative imaging) enhances understanding of the anatomic orientation and sites of margin sampling during pathologic processing. (p-value)	I feel confident in the treatment plans created using the 3D specimen map in addition to the current tools available at head & neck tumor board. (p-value)	I feel confident in locating the site of a positive margin using the 3D specimen map in addition to the current tools available at head & neck tumor board. (p-value)
**Years in Practice**	0.7	0.63	0.15	0.51	0.73	0.35
**Medical Specialty[1]**	0.23	0.23	0.24	0.17	0.18	0.41

[1]*Medical specialties were grouped into Head and Neck Surgeon (n=8), Medical/Radiation Oncology (n=8), Other (Dentistry, Pathology, Radiology, Speech Pathology)*

Supplementary Figure 1

Tumor Board Pre-Survey

Please complete the survey below.

Thank you!

---

1) Date

____

---

2) Level of Training

- ◯ Resident
- ◯ Fellow
- ◯ Faculty Provider

---

3) Specialty

- ◯ Surgeon
- ◯ Medical Oncology
- ◯ Radiation Oncology
- ◯ Pathology
- ◯ Radiology
- ◯ Speech Language Pathology
- ◯ Dentistry

---

4) The surgery, pathology, medical oncology, radiation oncology, and radiology teams are able to easily communicate and understand tumor characteristics for treatment planning at head & neck tumor board.

Strongly Disagree — Neutral — Strongly Agree

(Place a mark on the scale above)

---

5) The current tools used for discussion at head & neck tumor board (ie. operative reports, pathology reports, verbal discussions among the multidisciplinary team, pre and post-operative imaging) are adequate to understand the tumor size and characteristics.

Strongly Disagree — Neutral — Agree

(Place a mark on the scale above)

---

6) The current tools used for discussion at head & neck tumor board (ie. operative reports, pathology reports, verbal discussions among the multidisciplinary team, pre and post-operative imaging) are adequate to understand the anatomic orientation of the specimen and sites of margin sampling during pathologic processing.

Strongly Disagree — Neutral — Agree

(Place a mark on the scale above)

---

7) I feel confident in the treatment plans created using the current tools available at head & neck tumor board.

Strongly Disagree — Neutral — Strongly Agree

(Place a mark on the scale above)

---

8) I feel confident in locating the site of a positive margin using the current tools available at head & neck tumor board.

Strongly Disagree — Neutral — Strongly Agree

(Place a mark on the scale above)

Supplementary Figure 2

MEDREALITY

SCAN ME

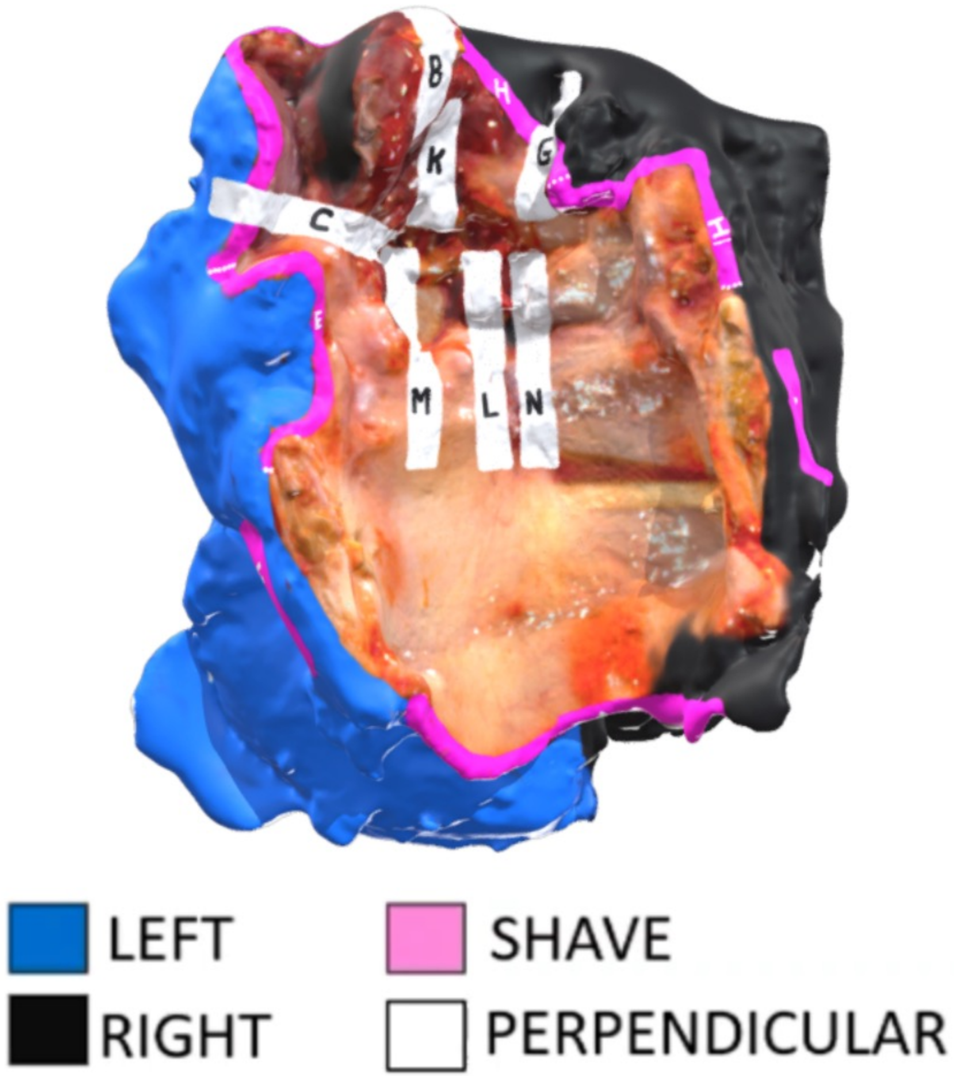

Supplementary Figure 3

MEDREALITY

SCAN ME

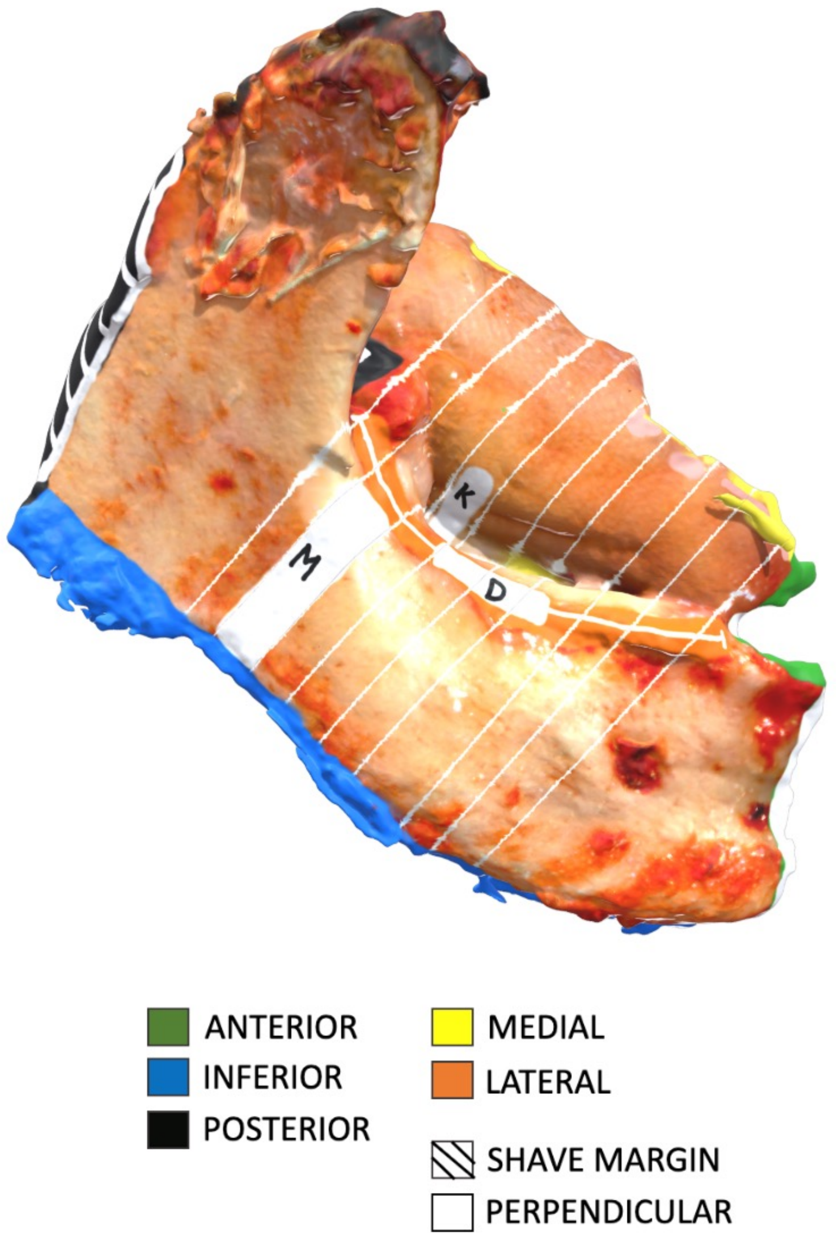

Supplementary Figure 4

MEDREALITY

SCAN ME

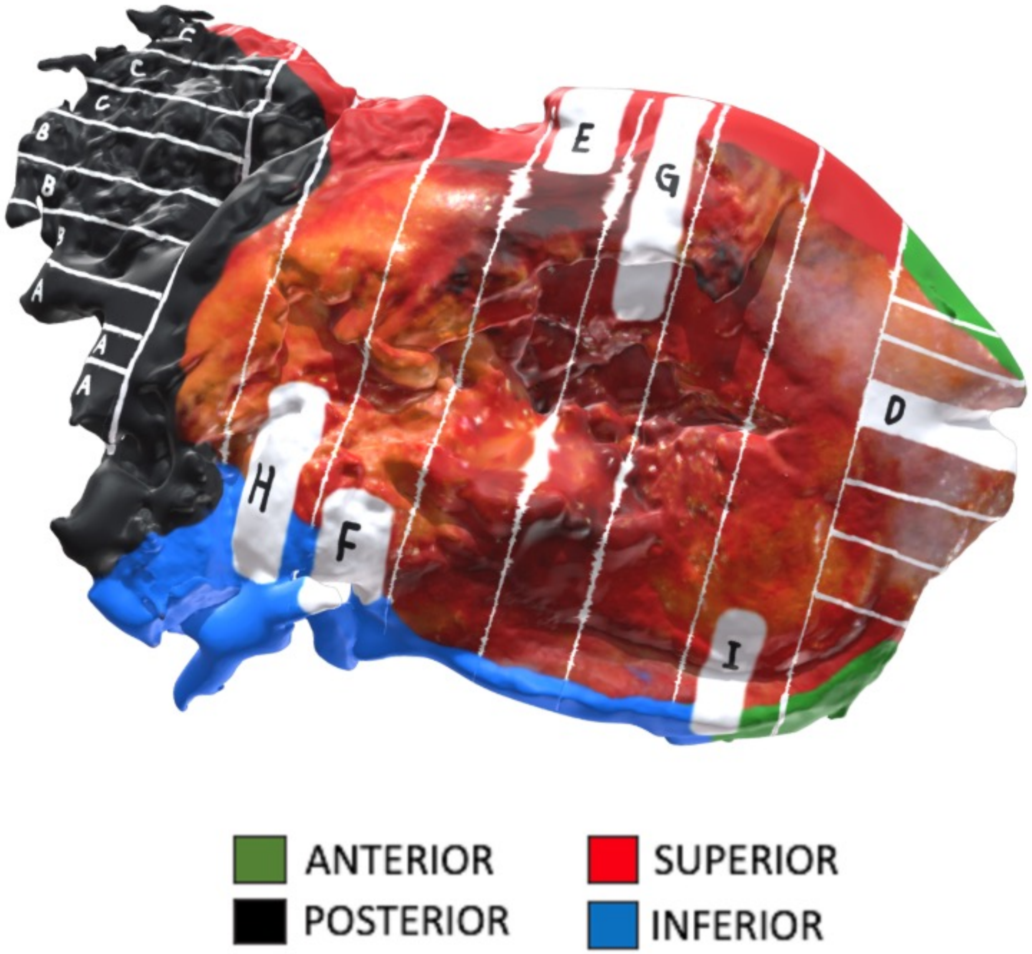

Supplementary Figure 5

MEDREALITY

SCAN ME

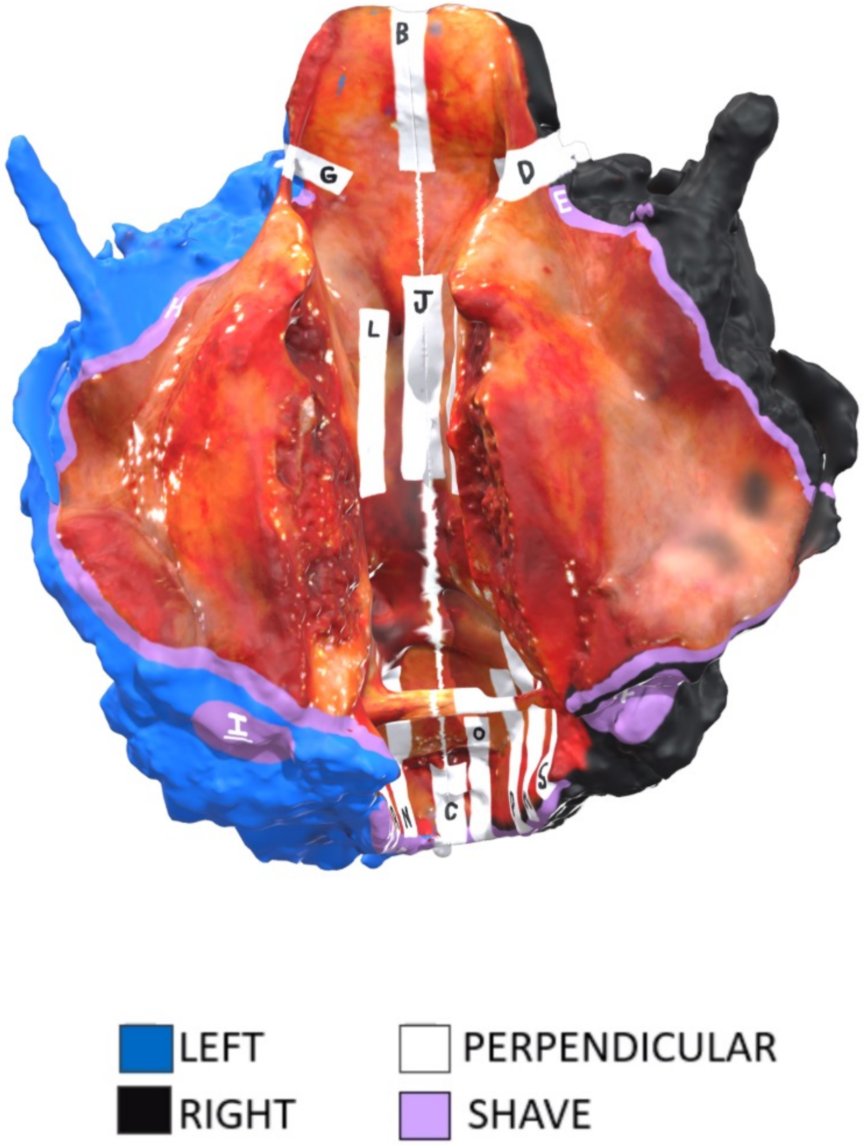

Supplementary Figure 6

MEDREALITY

SCAN ME

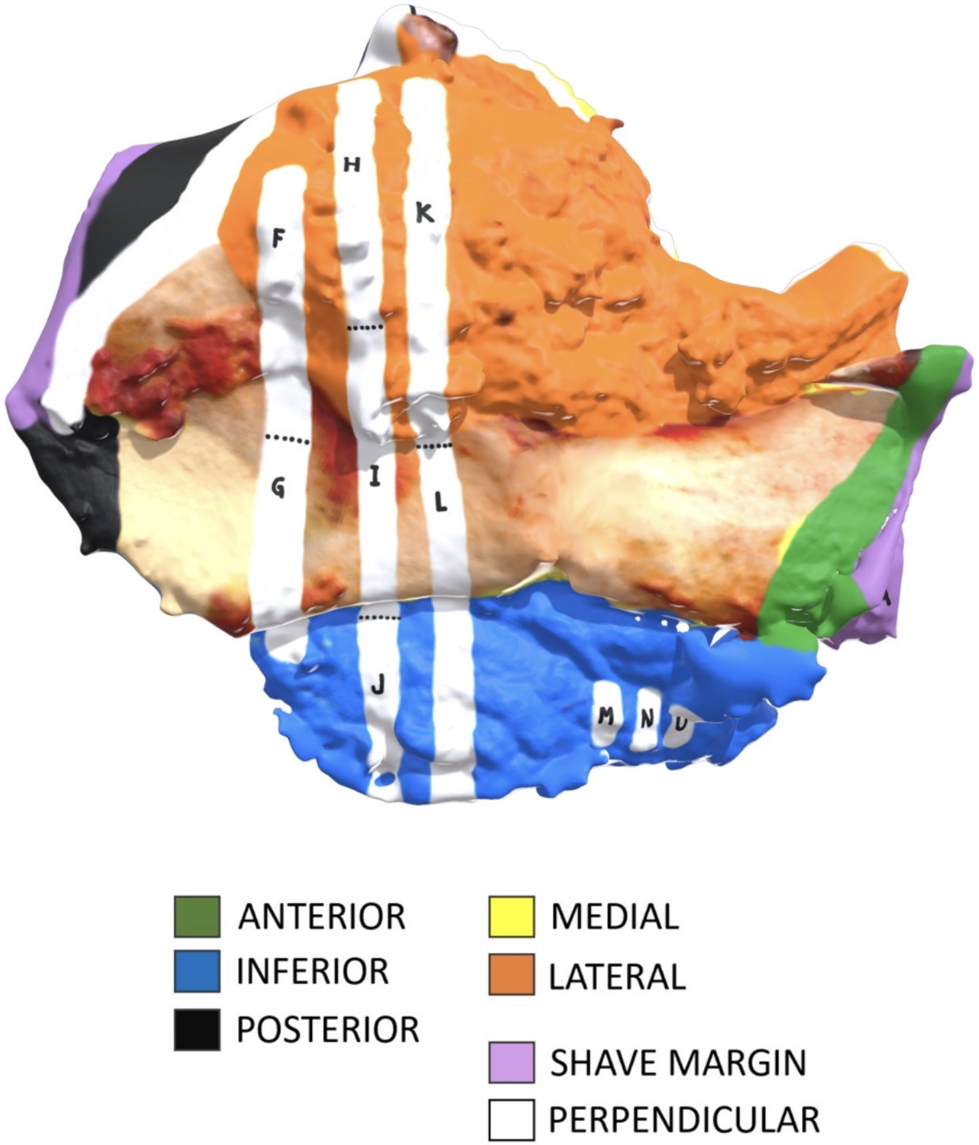

Supplementary Figure 7

SCAN ME

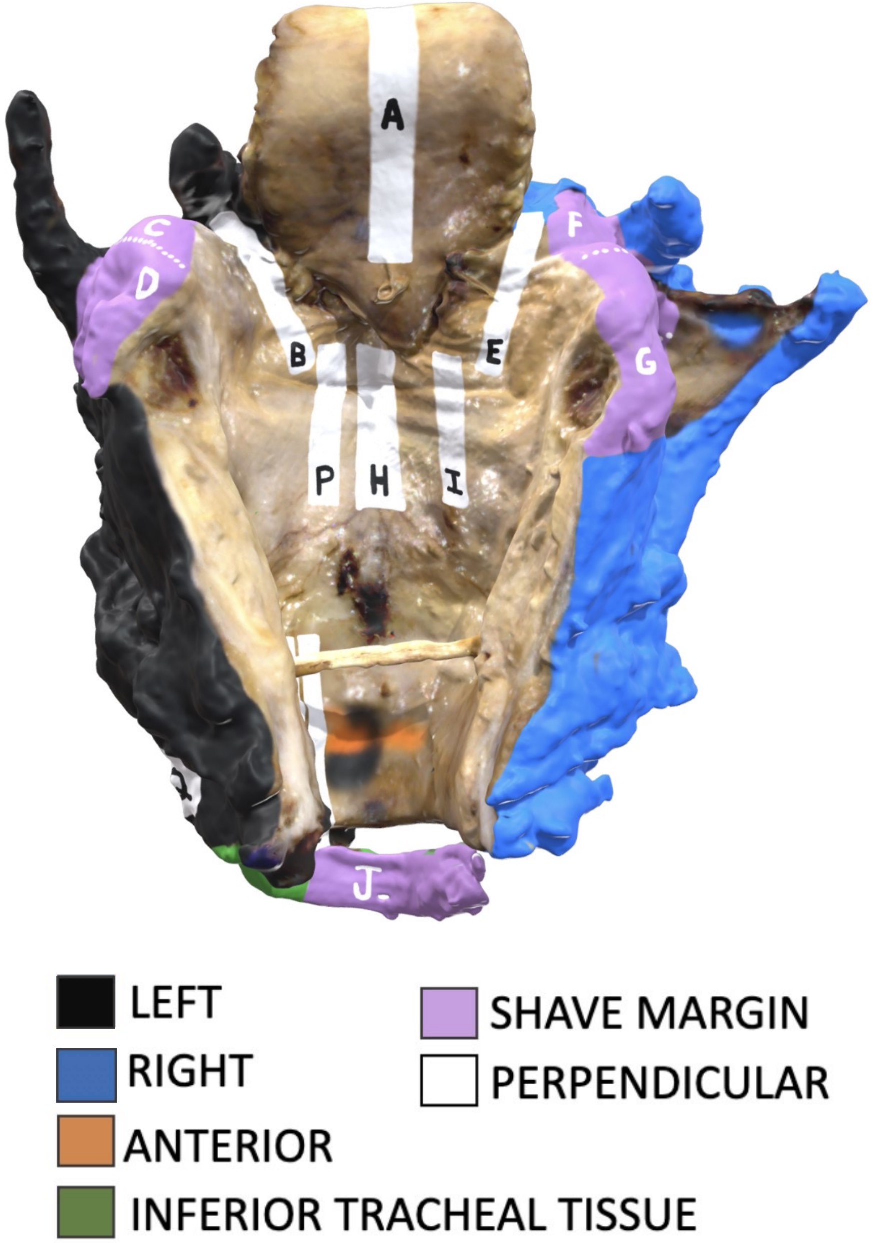

Supplementary Figure 8

MEDREALITY

SCAN ME

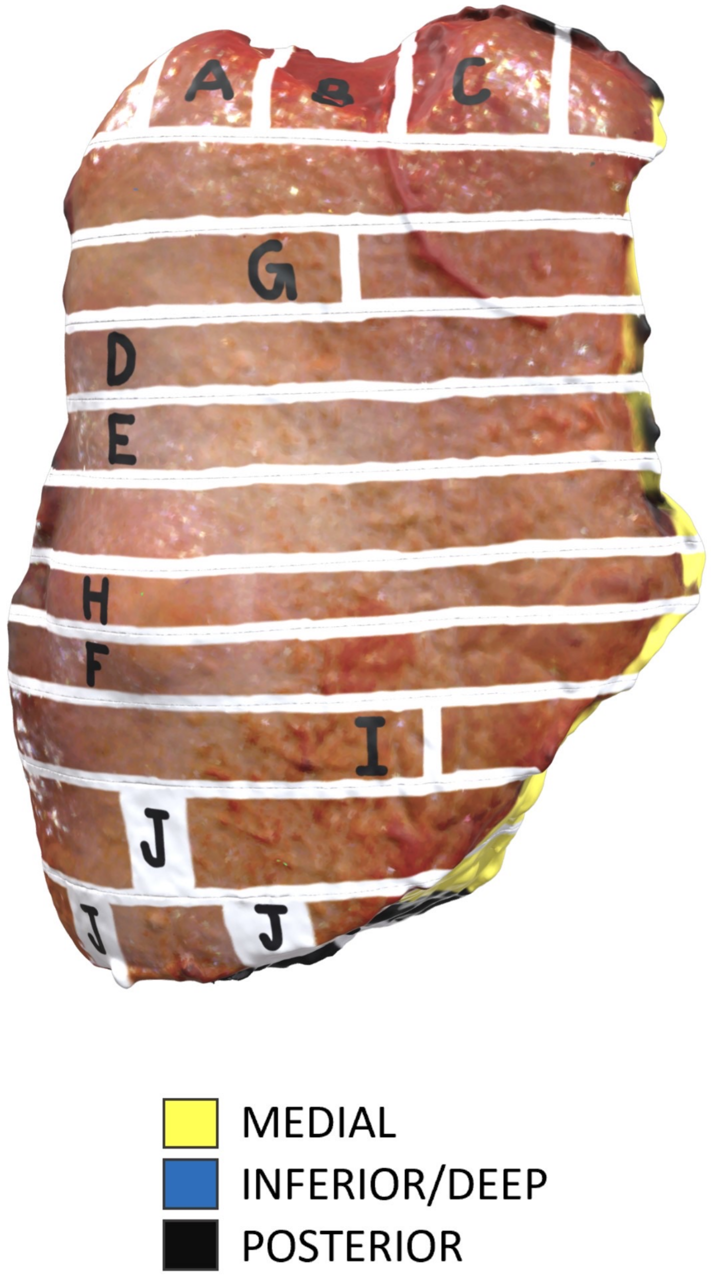

Supplementary Figure 9

MEDREALITY

SCAN ME

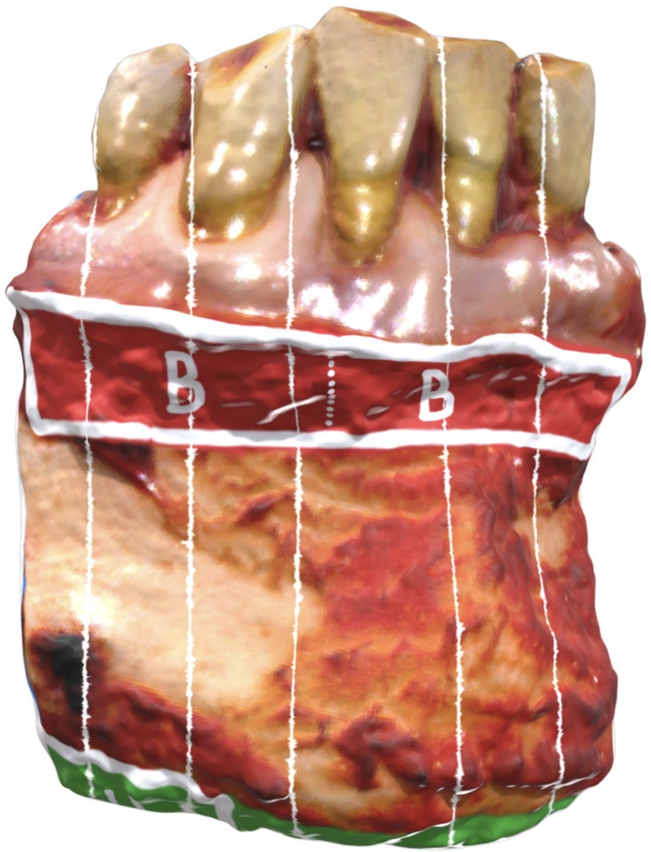

ANTERIOR
POSTERIOR
DEEP
RIGHT
LEFT
FROZEN
SHAVE (white outline, white letter)
A PERP (white box, black letter)

Supplementary Figure 10

MEDREALITY

SCAN ME

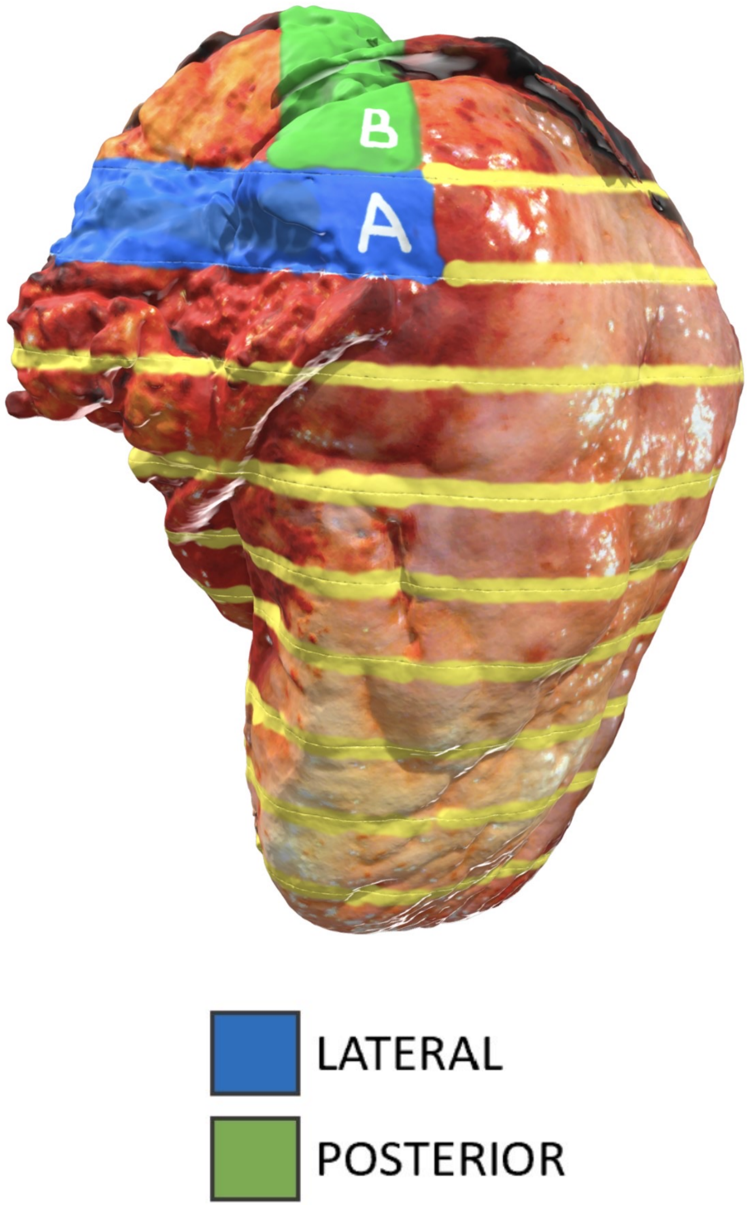

*This model represents the specimen based approach to the surgical specimen, not full grossing. Colored labels refer to lateral and posterior margins selected for analysis.

Tumor Board Post-Survey

Please complete the survey below.

Thank you!

1) Date

____

2) Level of Training

◯ Resident
◯ Fellow
◯ Faculty Provider

3) Specialty

◯ Surgeon
◯ Medical Oncology
◯ Radiation Oncology
◯ Pathology
◯ Radiology
◯ Speech Language Pathology
◯ Dentistry

4) The 3D specimen maps presented at head & neck tumor board are high quality.

Strongly Disagree — Neutral — Agree

(Place a mark on the scale above)

5) The surgery, pathology, medical oncology, radiation oncology, and radiology teams are able to easily communicate and understand tumor characteristics for treatment planning at head & neck tumor board.

Strongly Disagree — Neutral — Strongly Agree

(Place a mark on the scale above)

6) The 3D specimen map in addition to the current tools used for discussion at head & neck tumor board (ie. operative reports, pathology reports, verbal discussions among the multidisciplinary team, pre and post-operative imaging) enhances understanding of the tumor size and characteristics.

Strongly Disagree — Neutral — Strongly Agree

(Place a mark on the scale above)

7) The 3D specimen map in addition to the current tools used for discussion at head & neck tumor board (ie. operative reports, pathology reports, verbal discussions among the multidisciplinary team, pre and post-operative imaging) enhances understanding of the anatomic orientation and sites of margin sampling during pathologic processing.

Strongly Disagree — Neutral — Strongly Agree

(Place a mark on the scale above)

8) I feel confident in the treatment plans created using the 3D specimen map in addition to the current tools available at head & neck tumor board.

Strongly Disagree — Neutral — Strongly Agree

(Place a mark on the scale above)

9) I feel confident in locating the site of a positive margin using the 3D specimen map in addition to the current tools available at head & neck tumor board.

Strongly Disagree Neutral Strongly Agree

(Place a mark on the scale above)

10) I believe that 3D specimen maps can be integrated into the workflow of head & neck tumor board.

Strongly Disagree Neutral Strongly Agree

(Place a mark on the scale above)
